# MICROBIOTA INSIGHTS IN CLOSTRIDIUM DIFFICILE INFECTION AND INFLAMMATORY BOWEL DISEASE

**DOI:** 10.1080/19490976.2020.1725220

**Published:** 2020-03-04

**Authors:** C. Rodríguez, E. Romero, L. Garrido-Sanchez, G. Alcaín-Martínez, RJ. Andrade, B. Taminiau, G. Daube, E. García-Fuentes

**Affiliations:** a Instituto de Investigación Biomédica de Málaga-IBIMA, Málaga, Spain; b Unidad de Gestión Clínica de Aparato Digestivo, Hospital Universitario Virgen de la Victoria, Málaga, Spain; c Unidad de Gestión Clínica de Endocrinología y Nutrición, Hospital Universitario Virgen de la Victoria, Málaga, Spain; d Department of Medicine and Dermatology, Universidad de Málaga, Málaga, Spain; e Centro de Investigación Biomédica en Red de Enfermedades Hepáticas y Digestivas (CIBERehd), Málaga, Spain; f Fundamental and Applied Research for Animals & Health (FARAH), Department of Food Microbiology, Faculty of Veterinary Medicine, University of Liège, Liège, Belgium

**Keywords:** *C. difficile*, inflammatory bowel disease, microbial communities, microbial metabolites, interactions

## Abstract

Inflammatory bowel disease (IBD) is characterized by chronic intestinal inflammation that includes Crohn´s disease (CD) and ulcerative colitis (UC). Although the etiology is still unknown, some specific factors have been directly related to IBD, including genetic factors, abnormal intestinal immunity, and/or gut microbiota modifications. Recent findings highlight the primary role of the gut microbiota closely associated with a persistent inappropriate inflammatory response. This gut environment of dysbiosis in a susceptible IBD host can increasingly worsen and lead to colonization and infection with some opportunistic pathogens, especially *Clostridium difficile. C. difficile* is an intestinal pathogen considered the main cause of antibiotic-associated diarrhea and colitis and an important complication of IBD, which can trigger or worsen an IBD flare. Recent findings have highlighted the loss of bacterial cooperation in the gut ecosystem, as well as the pronounced intestinal dysbiosis, in patients suffering from IBD and concomitant *C. difficile* infection (CDI). The results of intestinal microbiota studies are still limited and often difficult to compare because of the variety of disease conditions. However, these data provide important clues regarding the main modifications and interrelations in the complicated gut ecosystem to better understand both diseases and to take advantage of the development of new therapeutic strategies. In this review, we analyze in depth the gut microbiota changes associated with both forms of IBD and CDI and their similarity with the dysbiosis that occurs in CDI. We also discuss the metabolic pathways that favor the proliferation or decrease in several important taxa directly related to the disease.

## Introduction

*Clostridium (Clostridioides) difficile* is a worldwide public health concern and is considered the major cause of antibiotic-associated infections in healthcare settings. It is responsible for serious outbreaks of hospital-acquired infections and for several sporadic diarrheas in the community. The pathogen is a sporulating, strictly anaerobic bacterium, and transmission occurs mainly by the fecal-oral route. Intestinal colonization and toxin production are necessary to trigger the infection; therefore, the disease is strongly related to the disruption of the gut microbiome^1^.

Inflammatory bowel disease (IBD) is a chronic disease of mainly the intestinal tract that includes ulcerative colitis (UC) and Crohn´s disease (CD). UC is a diffuse, continuous, and nonspecific inflammation of the colonic mucosa proximal to the rectum. Crohn´s disease is a chronic granulomatous inflammation that affects the entire digestive tract, especially the ileocaecum and perianal regions. While the cause of both disease forms is still unknown, some specific factors have been directly related to IBD, including genetic factors, abnormal intestinal immunity, and gut microbiota modifications directly caused by diet or infections.^2^

Patients suffering from IBD are particularly susceptible to *C. difficile* infection (CDI), with an increase in morbidity and mortality.^3^ Even if it is not clear if IBD itself or disease activity is an independent risk factor for CDI,^3,4^ further predisposing and specific conditions have been suggested in these patients,^3^ including colectomy and ileal-anal pouch anastomosis,^5^ nonsteroidal anti-inflammatory drugs,^6^ proton pump inhibitors, and other immunosuppressant treatments.^7^ Recurrence of CDI is common in IBD,^8^ and in the most complicated cases, only gut ecosystem restoration by fecal microbiota transplantation can help to break the cycle of recurrence.^9^ In the last decade, the availability of new omic technologies has allowed the investigation of gut microbial communities to identify whether any change in the bacterial composition is involved in CDI or an IBD flare.

This review analyses all the latest findings about the specific role of the gut microbiota composition in intestinal inflammation and infection. We will also focus on the decrease in gut diversity and its causative role in the development of CDI in patients suffering from IBD. Finally, we will link all these modifications in the gut with the production of microbial metabolites and their role in the worsening of CDI and IBD.

## A brief history of concomitant CDI and IBD

**Figure 1.**
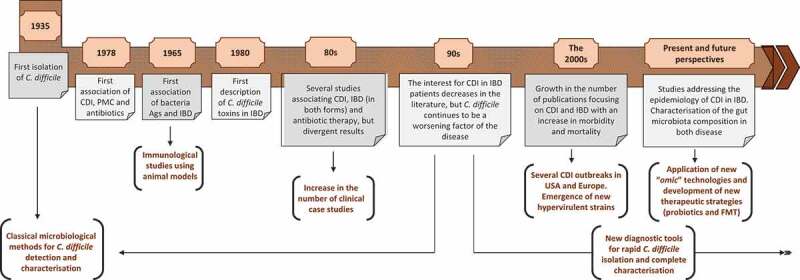
Early history of concomitant CDI and IBD.

The history of *C. difficile* dates back to 1935 (Figure 1), when the bacterium was isolated for the first time from the feces of breastfed infants.^10^ Despite the interest that the bacterium aroused in the following years, it was not until 1978 that it was first associated with pseudomembranous colitis and previous antibiotic therapy.^11,12^ First, immunological studies of ulcerative colitis observed the increase in anti-colon antibody titers due not only to chronic colon alteration, but also to unrelated gastrointestinal diseases, such as those caused by *C. difficile, Staphylococcus aureus*, Forsman antigen or *Escherichia coli* (*E. coli*) 014. These early findings showed that some antigens, especially those from *E. coli* 014, may contribute to colon autoimmunity in ulcerative colitis through disruption of tolerance.^13^ In 1980, two studies that were published in the literature almost simultaneously described for the first time the presence of *C. difficile* toxins in patients with IBD during a symptomatic relapse and suggested the association of these toxins with further complications in chronic disease or even with an IBD flare.^14,15^ In the following years, several other reports documented the possible association between CDI and IBD, and different studies began to investigate more specifically the role of *C. difficile* and its toxins, differentiating patients with UC and CD, although the results were not always the same or conclusive.^16–18^ Some studies directly associated the bacterium with toxic megacolon, acute relapses of IBD and/or hospital admissions.^19,20^ However, other further studies began to question the role of *C. difficile* in both forms of IBD, suggesting that the bacterium could be a part of the bowel gut, without specific cytopathic effects in the intestinal tissues of these patients, and that it would be relevant in only specific cases with previous antimicrobial therapies.^21,22^ The diversity in *C. difficile* detection methods used at the time varied greatly among the different studies, and they were not always as sensitive as needed.^23^ Furthermore, clinical evidence of the role of *C. difficile* in IBD patients was scarce.^24^

During the 1990s, a few studies were published reporting the presence of toxigenic *C. difficile* in the feces of IBD patients, with a prevalence that varied strongly up to 32%, and they described an exacerbation of the disease with the presence of the bacterium.^25,26^ However, it was not until the 2000s when there is an important growth in the number of publications that focused repeatedly on the impact of CDI in patients with IBD.^27–29^ This growing interest concurred with several outbreaks of CDI in hospitals in Europe and in the United States, and it is at this moment when *C. difficile* went from being an intestinal pathogen associated with antibiotic therapy to being the most important cause of nosocomial diarrhea in humans.^30^ Therefore, it seems logical that the IBD population was also increasingly affected by this pathogen, as reflected in the literature, with significant morbidity and mortality.^31^ Other additional but not mutually exclusive possibilities to explain the increase in the incidence of CDI in IBD patients in the last two decades are the evolution of the detection methods for the bacterium, the rapid diagnosis of the infection and a change in the epidemiology, with the emergence of new, best adapted, hypervirulent, and multidrug-resistant strains.^32,33^

In addition to epidemiological and clinical studies addressing the impact of CDI in IBD patients, in the last decade, several studies have investigated the interplay between the gut microbiota and disease. Advances in culture-independent molecular methods have allowed the identification of these bacterial populations present in the gut at each phase of the disease, which is followed by the use of new promising therapies, such as fecal microbiota transplantation or diet strategies (including prebiotics and probiotics), to successfully treat both of the diseases.

## Gut microbial community imbalances in human IBD

In IBD patients, intestinal tissue alteration by bacteria and/or inflammation results in a favorable environment with readily available nutrient sources leading to important perturbations in the normal composition of gut bacteria, in their functions, and finally in their metabolism. These alterations are potential precursors of other concomitant infections, including not only CDI but also other bacterial enteric pathogens, e.g., cytomegalovirus, enteroviruses, *Mycoplasma pneumoniae*, and upper respiratory viruses, and *Entamoeba histolytica*, among others. This perturbation finally triggers an important relapse or exacerbation of IBD symptoms.^34^

The results of intestinal microbiota studies are often difficult to compare due to different factors, including patient´s variability or the analysis methods and techniques used. Different sequencing technologies, annotation tools, and statistical analysis have been developed to study the microbial diversity and changes in the gut ecosystem. Several recent reviews on metagenomics (from sampling to data analysis) are already available in the literature^35^ and they show the need for a standardization of analysis techniques and workflows, in order to avoid variability in the results related to the methodology of the study.

Regarding patients, the variety of disease treatments (such as surgical interventions, anti-inflammatory drugs, biologics, immunosuppressant treatments, corticosteroids, proton pump inhibitors or antibiotics) and the demographical characteristics of the study population (gender, age, other overlapping syndromes, diet, smoking history, etc.) could have a direct impact on the results obtained.^36–39^ For example, it was reported that liver diseases could be the primary factors associated with disease-specific dysbiotic influences of IBD patients.^36^ Disease phenotypes (including IBD extent or activity) have also been associated with important changes in the gut mucosa, especially in CD,^39^ while other study observed no significant changes in the gut microbiota of UC patients after the use of biologic treatment (infliximab, adalimumab, or golimumab).^36^ Other described factors that influence the intestinal bacterial structure are the sample origin (stool or biopsy), as well as the biopsy location, which can induce other changes.^37,38^ In this context, it has been observed that the microbial imbalance due to intestinal inflammation is not always reflected in the lumen or in the stool. Therefore, a complementary analysis of tissue biopsies would be necessary to identify disease biomarker signals.^40^

Despite all these interindividual, sample, or methodological factors, the recovery of consistent changes in the bacterial composition, which are repeatedly reported in different studies investigating IBD patients, can highlight disease-specific bacterial signatures. It has also been suggested that changes in gut bacterial communities are not only a consequence of inflammation but also possible primary factors in disease.^37^ These changes could be interpreted as promising biomarkers, noninvasive diagnostic tools or new therapeutic approaches.

### Ulcerative colitis

General disruption of gut homeostasis in patients with UC is characterized by a depleted mucous layer (loss of mucus-containing goblet cells), a decrease in microbial alpha diversity,^36,41^ an increase in bacterial penetration, and an exaggerated Ig response, especially for IgA and IgG.^37^ Recently, all these changes were observed in both inflamed and non-inflamed intestinal sections of pediatric UC patients, suggesting that they are not a result of inflammation but rather precede (and probably promote) the disease.^37^ However, it remains unclear if the decrease in bacterial diversity is the cause or the result of the depleted mucus layer.^37^ Differences in bacterial diversity have been found when UC groups are compared with control individuals.^36,41,42^ The global microbiota composition was shifted by the presence of UC, with a reduced number of species and diminished richness and evenness, with an alteration in the community composition and structure.^41^ However, these findings can also be observed in both forms of IBD.^40,41.^

Specific gut microbiota signatures have been detected in patients suffering from UC. These changes include a decrease in the abundance of the phylum Verrucomicrobia^36^ or a decrease in the family Leuconostocaceae, the latter being known as acetate and lactate producers^38.^ There is not a clear consensus proportion of the genus *Bacteroides* within the phylum Bacteroidetes. While some studies in the literature reported significant reduction in *Bacteroides*,^42^ further studies found that this group is increased in UC patients^43^ and directly associated with the degradation of acid mucin as a carbon source in the colon, with an exaggerated inflammatory response and with colitis.^37^ Regarding the Clostridia class (phylum Firmicutes), we found some differences between families and genera, especially for the Clostridiaceae, Ruminococcaceae, and Lachnospiraceae families.^36–39,44^ In UC, studies have reported a reduction in the proportions of the genus *Coprococcus* and some species of genus *Roseburia*, along with other genera and species belonging to the family Lachnospiraceae.^36,39^ In contrast, for the Clostridiaceae family, some species seem to be in increased proportions in these patients,^37^ like *Clostridium symbiosum*, while a reduction in the abundance of other groups, such as *Clostridium colinum* and *Clostridium subcluster XIVab*, has also been described.^36,42^ Additionally, in the order Clostridiales, a decrease in the genus *Phascolarctobacterium* was linked to the presence of colonic inflammation regardless of the UC phenotype^36,38^ (Tables 1 and 2).

**Table 1.** Main changes detected in the gut microbiota of patients suffering from ulcerative colitis and Crohn´s disease. Bacteria for which a reduction in relative proportions has been detected.

Taxa depletion in the gut of patients with IBD								
Phylum level	Class level	Order level	Family level	Genus level	Species level	IBD disease^Ψ^	Type of sample^Ψ^	Ref
Actinobacteria	-	-	-	-	-	*CD, UC*	*Biopsy*	45
	Actinobacteria	Actinomycetales	Actinomycetaceae	-	-	*UC*	*Stools*	46
		Bifidobacteriales	Bifidobacteriaceae	-	-	*CD*	*Stools*	47
				*Bifidobacterium*	-	*CD*	*Biopsy* *Stools*	38,40,46,48
		Coriobacteriales	Coriobacteriaceae	-	-	*UC*	*Stools*	46
Bacteroidetes	-	-	-	-	-	*CD, UC*	*Biopsy* *Stools*	49
	Bacteroidia	Bacteroidales	-	-	-	*CD*	*Stools*	47,50
			Bacteroidaceae	-	-	*CD*	*Biopsy* *Stools*	37,40
				*Bacteroides*	-	*CD, UC*	*Biopsy*	49
					*B. fragilis*	*CD*	*Stools*	51
					*B. ovatus*	*UC*	*Biopsy*	37
					*B. thetaiotaomicron*	*CD, UC*	*Biopsy*	52
					-	*CD*	*Biopsy* *Stools*	53-55
			Barnesiellaceae			*CD, UC*	*Biopsy*	37
			Odoribacteraceae			*CD*	*Biopsy*	37
				*Odoribacter*	-	*CD, UC*	*Biopsy* *Stools*	37,56
					*O. splanchnus*	*CD*	*Biopsy* *Stools*	38
			Porphyromonadaceae	-	.	*CD, UC*	*Biopsy*	37
				*Parabacteroides*	*P. distasonis*	*CD*	*Stools*	56
						*CD, UC*	*Biopsy*	49
					*P. merdae*	*CD, UC*	*Biopsy*	37
			Prevotellaceae	*Prevotella*	-	*CD*	*Biopsy* *Stools*	50,56
					*P. copri*	*CD, UC*	*Stools*	57
			Rikenellaceae	-	-	*CD, UC*	*Biopsy* *Stools*	37,49
				*Alistipes*	-	*CD, UC*	*Biopsy* *Stools*	52,56
					*A. onderdonkii*	*CD, UC*	*Biopsy*	37
					*A. finegoldii*	*CD*	*Biopsy*	37
					*A. putredinis*	*CD*	*Biopsy*	37
Firmicutes	-	-	-	-	-	*CD, UC*	*Biopsy*	45,49,58
	Bacilli	Lactobacillales	Lactobacillaceae	*Lactobacillus*	-	*CD, UC*	*Biopsy*	55
			Leuconostocaceae	-	-	*UC*	*Biopsy*	38
	Erysipelotrichia	Erysipelotrichales	Erysipelotrichaceae	-	-	*CD*	*Biopsy* *Stools*	38,46,47
	Clostridia	Clostridiales	-	-	-	*CD, UC*	*Biopsy* *Stools*	40,46–48,58
			Clostridiaceae	-	-	*CD, UC*	*Stools Biopsy*	38,39,46,59
				*Clostridium*	*C. coccoides*	*CD*	*Stools*	51,54
					*C. colinum*	*UC*	*Stools*	36
					*C. nexile*	*CD, UC*	*Biopsy*	52
					*C. leptum*	*CD*	*Stools*	51,54
				*Butyricicoccus*	*B. pullicaecorum*	*UC*	*Stools*	36
			Christensenellaceae	-	-	*CD, UC*	*Biopsy* *Stools*	46
			*Dehalobacteriaceae*	-	-	*CD, UC*	*Stools*	46
			Eubacteriaceae	*Eubacterium*	-	*CD, UC*	*Biopsy* *Stools*	55,56
					*E. rectale*	*UC*	*Stools*	60
			Lachnospiraceae	-	-	*CD, UC*	*Biopsy* *Stools*	38,40,46,49
				*Blautia*	-	*CD*	*Biopsy*	40
				*Coprococcus*	-	*CD*	*Biopsy* *Stools*	40,43,50,56
				*Coprococcus*	-	*CD. UC*	*Biopsy*	49
					*C. catus*	*UC*	*Stools*	36
				*Dorea*	*D. formicigenerans*	*CD, UC*	*Stools*	61
					-	*CD*	*Biopsy* *Stools*	40,56
				*Roseburia*	-	*CD, UC*	*Biopsy* *Stools*	36,38–40,45,46,50,56
					*R. hominis*	*CD, UC*	*Biopsy* *Stools*	61,62
			*Mogibacteriaceae*	-	-	*CD, UC*	*Stools*	46
			*Peptococcaceae*	-	-	*CD*	*Stools*	46
			*Peptostreptococcaceae*	-	-	*CD*	*Stools*	46
			Ruminococcaceae	-	-	*CD, UC*	*Biopsy* *Stools*	38,40,46,49
				*Oscillospira*	-	*CD, UC*	*Stools*	49
				*Ruminococcus*	-	*CD*	*Stools*	46,56
					*R. obeum*	*CD, UC*	*Stools*	61
				*Faecalibacterium*	-	*CD*	*Biopsy* *Stools*	38-40,46,48-50
					*F. prausnitzi*	*CD, UC*	*Biopsy* *Stools*	36,45,49,54,57,60,62-66
				*Ruminococcus*	-	*CD*	*Biopsy*	39,40
					*R. gnavus*	*UC*	*Stools*	36
			Veillonellaceae	*Phascolarctobacterium*	-	*CD, UC*	*Biopsy* *Stools*	36,38
Euryarchaeota	Methanobacteria	Methanobacteriales	Methanobacteriaceae	-	-	*UC*	*Stools*	46
Proteobacteria	Deltaproteobacteria	Desulfovibrionales	Desulfovibrionaceae	*Desulfovibrio*	*D. simplex*	*CD*	*Biopsy*	37
					*D. vulgaris*	*CD*	*Biopsy*	37
Ternericutes	Mollicutes	-	-	-	-	*UC*	*Biopsy* *Stools*	46
Verrucomicrobia	-	-	-	-	-	*CD, UC*	*Stools*	49
	Verrucomicrobiae	Verrucomicrobiales	Verrucomicrobiaceae	*Akkermansia*	*A. muciniphila*	*CD, UC*	*Biopsy* *Stools*	36,56,67

**Ψ** Group of bacteria described *in at least* one form of IBD disease (ulcerative colitis or Crohn´s disease) and in at least one type of sample (biopsy or stools).

**Table 2.** Main changes detected in the gut microbiota of patients suffering from ulcerative colitis and Crohn´s disease. Bacteria for which an increase in relative proportions has been detected.

TAXA INCREASE IN THE GUT OF PATIENTS WITH IBD								
Phylum level	Class level	Order level	Family level	Genus level	Species level	IBD disease^Ψ^	Type of sample^Ψ^	Ref
Actinobacteria	-	-	-	-	-	*CD, UC*	*Biopsy*	52
	Actinobacteria	Acidimicrobiales	Acidimicrobidae	-	-	*CD, UC*	*Biopsy*	52
		Actinomycetales	Actinomycetaceae	*Actinomyces*	*A. oxydans*	*CD, UC*	*Biopsy*	52
			Nocardioidaceae	*Nocardioioides*	-	*CD, UC*	*Biopsy*	52
		Bifidobacteriales	Bifidobacteriaceae	*Bifidobacterium*	*–*	*CD*	*Biopsy*	50
					*B. adolescentis*	*CD*	*Stools*	68
					*B. breve*	*UC*	*Stools*	61
Bacteroidetes	Bacteroidia	Bacteroidales	Bacteroidaceae	*Bacteroides*	-	*CD, UC*	*Biopsy* *Stools*	37,43,46,48,58,59,69
					*B. distasonis*	*CD*	*Stools*	51
			-	-	-	*CD, UC*	*Stools*	43,46
			Porphyromonadaceae	*Porphyromonas*	-	*UC*	*Biopsy*	43
				*Parabacteroides*	-	*CD*	*Stools*	46
					*P. distasonis*	*CD*	*Stools*	68
			Prevotellaceae	*Prevotella*	-	*CD, UC*	*Biopsy* *Stools*	43,51
					*P. copri*	*CD, UC*	*Stools*	68
			Rikenellaceae	-	-	*UC*	*Stools*	46
Firmicutes	Bacilli	-	-	-	-	*CD, UC*	*Biopsy* *Stools*	43,46,69
		Bacillales	Bacillaceae	*Bacillus*	*B. licheniformis*	*CD, UC*	*Biopsy*	52
		Lactobacillales	Aerococcaceae	-	-	*CD*	*Stools*	_45_
			Lactobacillae	-	-	*UC*	*Stools*	46
				*Lactobacillus*	-	*CD, UC*	*Stools*	40,49
			Enterococcaceae	-		*UC*	*Stools*	46
				*Enterococcus*	-	*CD*	*Stools*	40,56
			Streptococcaceae	*Streptococcus*	-	*CD, UC*	*Biopsy* *Stools*	40,49
		Staphylococcales	Gemellaceae			*CD*	*Stools*	47
				*Gemella*	*G. moribillum*	*CD*	*Stools*	40
	Clostridia	Clostridiales	Clostridiaceae	*Clostridium*	-	*CD, UC*	*Biopsy* *Stools*	38
					*C. bolteae*	*CD*	*Biopsy* *Stools*	62
					*C. clostridioforme*	*CD*	*Stools*	61
					*C. hathewayi*	*CD*	*Biopsy* *Stools*	62
					*C. symbiosum*	*UC*	*Stools*	61
					*C. ramosum*	*UC*	*Stools*	60
			Lachnospiraceae	-	-	*CD*	*Biopsy*	37,46
				*Lachnobacterium*	-	*UC*	*Stools*	46
				*Blautia*	-	*UC*	*Biopsy*	50
				*Roseburia*	-	*CD, UC*	*Biopsy* *Stools*	50,61
			Ruminococcaceae	-	-	*CD*	*Biopsy*	37
				*Faecalibacterium*	-	*CD*	*Biopsy*	50
				*Ruminococcus*	-	*CD, UC*	*Stools*	49
					*R. gnavus*	*CD, UC*	*Biopsy* *Stools*	43,50,61,62,67
					*R. torques*	*CD, UC*	*Biopsy* *Stools*	62,67
	Negativicutes	Selenomonadales	Acidaminocaccaceae	*Acidaminococcus*	-	*CD, UC*	*Biopsy*	45
		Vellionellales	Veillonellaceae	-	-	*CD, UC*	*Biopsy* *Stools*	40
				*Veillonella*	-	*CD*	*Stools*	56
					*V. dispar*	*CD, UC*	*Biopsy*	45
					*V. parvula*	*CD*	*Stools*	40
Fusobacteria	Fusobacteriia	Fusobacteriales	Fusobacteriaceae	-	-	*CD*	*Biopsy* *Stools*	40,47,69
				*Fusobacterium*	*F. nucleatum*	*CD*	*Stools*	40
Proteobacteria	-	-	-	-	-	*CD, UC*	*Biopsy* *Stools*	38,40,43,45,46,49,50,65,69
	Betaproteobacteria	Neisseriales	Neisseriaceae	-	-	*CD*	*Stools*	40
	Alphaproteobacteria	-	-		-	*CD, UC*	*Biopsy*	52
		Sphingomonadales	Sphingomonadaceae	*Sphingomonas*	-	*CD, UC*	*Biopsy*	52
				*Novosphingobium*	-	*CD, UC*	*Biopsy*	52
	Betaproteobacteria	Burkholderiales	-	-	-	*CD, UC*	*Biopsy* *Stools*	46
		Neisseriales	Neisseriaceae	-	-	*CD*	*Biopsy* *Stools*	40,47
	Gammaproteobacteria	-	-	-	-	*CD, UC*	*Biopsy*	52
		Enterobacteriales	Enterobacteriaceae	-	-	*CD, UC*	*Biopsy* *Stools*	9,37–40,45,46,49,68
				*Citrobacter*	-	*CD*	*Biopsy*	50
				*Escherichia*	-	*CD*	*Biopsy* *Stools*	38,50,56,61,62,69
					*E. coli*	*UC*	*Stools*	60
				*Klebsiella*	-	*CD*	*Stools*	56
				*Shigella*	-	*CD*	*Biopsy* *Stools*	38,69
		Pasteurellales	Pasteurellaceaea	-	-	*CD*	*Stools*	47
				*Actinobacillus*	-	*CD*	*Biopsy*	50
				*Haemophilus*	-	*CD*	*Biopsy*	40
		Pseudomonales	Pseudomonaceae	*Pseudomonas*	*P. straminea*	*CD, UC*	*Biopsy*	52

**Ψ** Group of bacteria described *in at least* one form of IBD disease (ulcerative colitis or Crohn´s disease) and in at least one type of sample (biopsy or stools).

### Crohn's disease

Most of the microbiota changes previously described in the gut microbiota of patients with UC are also observed in patients suffering from CD. For example, an increase in the proportion of the Enterobacteriaceae family is found in UC patients, which is also present in the gut of patients suffering from CD,^43,47,68^ especially regarding *Escherichia* and *Shigella* relative proportions, which are directly implicated in intestinal inflammation.^38,39,61^

**Figure 2.**
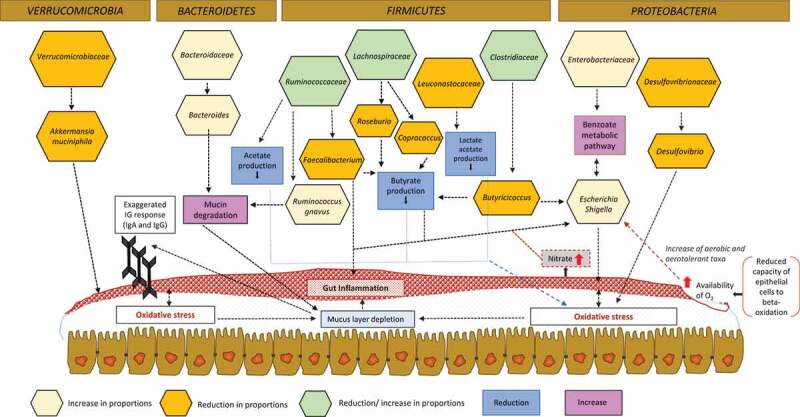
Changes in the gut microbiota communities of IBD patients.

In CD patients with ileal involvement, an important reduction in the proportions of Ruminococcaceae and *Faecalibacterium* has been reported in several studies (Table 1). They are recognized as acetate and butyrate producers, respectively, and therefore they contribute to creating an environment of oxidative stress in the intestine^38^ (Figure 2). In this context, while some bacteria seem to be associated with a specific disease phenotype, a decrease in Clostridiales is most likely present in all forms of CD^39,47,48,51,54,59,63^ with only a few exceptions.^61^ In contrast, there is no unanimity regarding the increase or decrease in Lachnospiraceae abundance in CD. While some studies reported an increase in the proportions of this family in the intestinal mucosa of patients with moderate activity,^37^ further studies reported a decrease in their proportions^38,40,52,61^ or an increase at the family level but a depletion in lower taxonomical levels.^46^ These findings may indicate an important correlation between intestinal dysbiosis and CD phenotype^39^ (Tables 1 and 2).

## Dysbiosis implications

At this point, we can observe that the available studies in the literature have described more similarities than dissimilarities in the gut environment of IBD patients, regardless of whether they suffer from CD or UC. Furthermore, the proportions of some phylum seem to vary in function of the compartments of the intestinal tract, notably in ileal, colonic tissue, and rectal tissue.^69^

The decrease in the alpha-diversity index and the increase or decrease in the different taxa in the gut have important functional implications for epithelium repair and inflammation regulation, playing a fundamental role in the course and worsening of the disease. The crosstalk of the bacterial groups in the gut ecosystem is a competitive, bidirectional, and dynamic process, which evolves in the function of the local environment. In turn, this environment directly depends on the available nutrients and the bacterial metabolites, which finally promotes differential bacterial growth. In IBD individuals, a decrease in basic biosynthesis has been observed, along with changes in several other metabolic processes (the biosynthesis of essential amino acids, cobalamin synthesis, purine and pyrimidine biosynthesis, acetogenesis to replace biomethanation, lipid catabolism, and phospholipid metabolism).^38–40^ Under these conditions, the levels of hydrogen are strongly reduced, promoting an increase in aerobic and aerotolerant taxa and exacerbating disease severity.^40^

### Proteobacteria phylum in IBD patient: enterobacteriaceae, pasteurellaceae, and desulfovibrionaceae families

Several studies have reported an increase in the Pasteurellaceae and/or Enterobacteriaceae families in patients with CD.^37–40,45-47,49,68^ Gut inflammation and chronic colitis have been further associated with an important increase of Enterobacteriaceae family^68^ and an oxidative stress in the gut. A recent study goes beyond and suggests Enterobacteriaceae as stool biomarkers in IBD.^45^ There are several metabolic changes that promote oxidative stress at the mucosal surface of IBD patients and favor an increased level or depletion of different taxa that use mucin as a primary energy source.^37,38^ Specifically, the increase in components of the benzoate metabolic pathway (aminobenzoate and fluorobenzoate degradation) seems to be directly associated with Enterobacteriaceae growth, virulence, and stress response.^40^ Bacteria such as *Salmonella* or enterohemorrhagic *E. coli* would take advantage of these redox stresses and therefore proliferate to a large extent. Indeed, in the ileum mucosa of CD patients and in the fecal samples of UC patients,^60^ high numbers of adherent and invasive *E. coli* have been found, as well as a high prevalence of antibodies directed against *E. coli* outer membrane porin C (OmpC) and flagellin. It seems that *E. coli* acts as an opportunistic pathogen and is directly implicated in the disease, with the induction of the production of cytokines, such as tumor necrosis factor α (TNFα) and IL8,^39^ and an increase in mucin degradation.

In contrast, the Desulfovibrionaceae family is reported in reduced proportions,^37^ with a negative correlation between physiological distress and its abundance.^70^ The genus *Desulfovibrio* degrades acidic mucin normally found in the colon. However, in CD patients, the mucus is mostly neutral, which can explain the reductions observed in these subjects.^37^

### Bacteroidetes pylum in IBD patients: prevotellaceae and bacteroidaceae families

In IBD patients, inflammation and colitis have been also associated with an increase of Prevotellaceae^43,51^ and Bacteroidaceae^46^ family. Within the latter, *Bacteroides* genus has been suggested as an IBD biopsy biomarker.^45^

There is an important increase in other bacterial-mediated processes, which in turns favor the proliferation of members of Bacteroidetes phylum. These processes include an increase in the metabolism of the sulfur amino acid cysteine, riboflavin metabolism, lipopolysaccharide production, glutathione biosynthesis, N-acetylgalactosamine phosphotransferase transporters, and virulence factor production. A decrease in proportions of *Bacteroides* genus has been reported in inflamed mucosa when compared with non-inflamed mucosa of patients suffering IBD,^49^ and have been identified as a predictor of relapse.^54^

### Firmicutes phylum in IBD patients: clostridiaceae, lachnospiraceae ruminococcaceae and veillonellaceae families

The oxidative stress previously described has also a direct impact in the phylum Firmicutes, with an increase in some mucolytic bacteria, especially *Ruminococcus torques* and *Ruminococcus gnavus*.^67^ A previous study showed differences in their abundance in the dysbiotic gut of CD and CU patients.^62^ Furthermore, *R. gnavus* along with *Clostridium hathewayi* and *Clostridium bolteae* have increased expression during dysbiosis, suggesting that they could have a role in the disease.^62^

On the other hand, a decrease in the abundance of butyrate-producing, hydrogen-utilizing bacteria and other taxa with anti-inflammatory activity, including *Faecalibacterium, Phascolarctobacterium* (Veillonellaceae family), and *Clostridia* clades IV and XIVa, especially the genera *Roseburia* (Lachnospiraceae family) and *Butyricicoccus* (Clostridiaceae family) have been observed (Table 1). Species of *Roseburia* are butyrate producers and acetate consumers and are associated with anti-inflammatory regulatory T cell production, while *Phascolarctobacterium* species are only succinate consumers. Both of these genera have been associated with a decrease in butyrate and propionate production in both forms of IBD.^36,38^ In relation to the genus *Faecalibacterium*, a low rate of *Faecalibacterium prausnitzii* have been identified as predictors of relapse.^54^ IBD inflamed mucosa presents a decrease in *F. prausnitzii* compared to non-inflamed mucosa.^49^ Furthermore, low abundance of *Faecalibacterium* in postoperative ileal mucosa has been associated with a higher risk of recurrence,^64^ and also associated with a massive increase of leukocytes in UC.^66^ This bacterium can metabolize host-derived polysaccharides (pectin, uronic acids) and other substrates (such as N-acetyl glucosamine) from the intestinal mucus for growth, and it is also described as an important anti-inflammatory commensal bacterium.^38^ The anti-inflammatory properties of *F. prausnitzii* have been associated with inhibition of the NF-κB pathway via protein production in intestinal epithelial cells, while a decrease in proportions of *Butyricicoccus pullicaecorum* seems to attenuate trinitrobenzene sulfonic acid (TNBS)-induced colitis in rats and to increased transepithelial resistance.^36^ This depletion of *Faecalibacterium* and *Butyricicoccus*, but also of *Ruminococcus hominis*, combined with alterations in bacterial products, especially butyrate,^53,57,62^ provides the appropriate conditions to allow *E. coli* to proliferate.^39^

### *Verrucomicrobia phylum*, akkermansia *abundance, and its impact on IBD*

Unlike for other mucolytic bacteria, some studies reported a reduction in the levels of *Akkermansia muciniphila* in both CD and UC patients and in the early onset of CD.^67,71^ It has been proposed that *A. muciniphila* could be responsible for exacerbated gut inflammation in IBD patients. However, a recent study using animal models did not find any correlation between short-term intestinal inflammation and the presence of the bacterium in the gut.^72^ *A. muciniphila* use mucus as a carbon, nitrogen, and energy source, and therefore, as a consequence of its metabolism, it produces short-chain fatty acids (acetate, propionate, 1,2-propanediol, and succinate).^73^ It is worth mentioning that short-chain fatty acids seem to be depleted in IBD patients.^74^ Furthermore, as specifically described for UC patients, the decrease in the abundance of the genus *Akkermansia* and the low abundance of *A. muciniphila* could affect the use of mucins as a carbon source by other symbiotic commensal bacteria,^36^ and it was proposed as a possible marker of dysbiosis.^75^ Reductions in *Akkermansia* genus were also described in the gut microbiota of patients with CD.^56^

## CDI and IBD: gut microbiota relationships and implications for disease treatment with fecal microbiota transplantation

Only a few studies have investigated the specific impact of CDI on the IBD microbiota. A previous study^76^ directly compared the gut microbiota of IBD patients with and without CDI disease. The authors found that in patients with both IBD and CDI, there was a loss of bacterial cooperation in the gut ecosystem as well as a more pronounced intestinal dysbiosis than in patients suffering from only IBD. Metabolite production is also altered in the inflamed gut, which is essential for several metabolic processes, as energy production and host immunity.^77^ Among these metabolites, butyrate has a fundamental role in maintaining the balance of the intestinal microbiota, with the preservation of the epithelial barrier and regulation of the immunity.^53,74^ Butyric acid, along with acetic acid and propionic acid are the end products of indigested carbohydrates in the intestine after bacterial fermentation. It was demonstrated that butyrate enhances the intestinal barrier function by facilitating the assembly of tight junctions.^78^ Butyrate producers supply energy to gut epithelial cells and therefore they protect against inflammation and infection.^79^ In the last decade, several studies have focused on the modification of the gut microbiota to successfully treat several intestinal diseases, including IBD and CDI. Among the various taxa investigated, it seems that positive results are obtained when the feces include the following taxa: *Clostridium* clusters *IV* and *XIVa*, which include the Ruminococcaceae and Lachnospiraceae families, respectively, and the genera *Roseburia, Oscillibacter, Blautia*, and *Dorea*. This selection of microbes has important underlying metabolic mechanisms, specially the production of butyrate in the gut.^80^

### Blautia *and* Dorea *genera: role in maintenance and recovery of gut homeostasis*

In concomitant IBD and CDI diseases, there is a specific reduction in two groups of bacteria, *Blautia* and *Dorea*. Members of *Blautia* (butyrate-producing bacterial species) are already significantly reduced in patients with only IBD, but it seems that the decrease in the relative proportions in the gut is more marked when the disease is aggravated with CDI.^76^ The *Blautia* genus (especially *Blautia obeum*) is selected among the bacterial species enriched in the fecal microbiota of healthy donors for fecal microbiota transplant (FMT) and was also found after successful restoration of the gut in patients with recurrent CDI.^81–85^ Furthermore, *in vitro* analysis has shown a negative correlation between the production of a bile metabolism enzyme (bile salt hydrolase, of which *Blautia* is one of the representative producers) and *C. difficile* germination.^86,87^ It was recently described that the bacterial strain *B. obeum* A2-162 produces a lantibiotic, nisin O, in the human gastrointestinal tract, which presents antimicrobial activity against both *Clostridium perfringens* and *C. difficile*.^88^ Therefore, its depletion would favor *C. difficile* colonization and infection in the IBD gut.

Regarding the *Dorea* genus, its depletion has been previously reported in studies investigating patients suffering from CDI and IBD separately.^40,79^ *Blautia* and *Dorea* have been described as major acetate producers in the normal gut, but it is hypothesized that they are replaced when CDI occurs.^79^ Recently, a study proposed a cocktail of bacteria to treat recurrent CDI, which includes *Blautia producta (Peptostreptococcus productus), E. coli* and *Clostridium bifermentans*. The authors demonstrate in their work that this bacteriotherapy could antagonize chronic relapse of CDI, which in turn inhibited the growth of *Bacteroides* species.^89^

### Undesirable increase of some taxa: proliferation of other pathogens and aggravating factors of disease

There are some taxa that seem to increase in abundance in patients suffering from IBD and CDI, including some species of *Clostridium, Enterococcus*, and *R. gnavus*,^76^ which have also been described to increase in patients with IBD (Table 2). High abundances of Proteobacteria and Enterobacteriaceae are often found in patients with recurrent CDI^82^ but also in those suffering from IBD.^37–40,46^ Within these two bacterial groups, some species are classified as important pathogens, such as *Salmonella* and enterohemorrhagic *E. coli*, which may take advantage of the intestinal conditions under dysbiosis to proliferate and worsen the disease.^39^ These intestinal conditions include altered oxygen availability and nitrate production. Under inflammatory conditions, epithelial cells reduce their capacity to undergo beta-oxidation, resulting in an increase in available oxygen and a reduction in hydrogen levels.^40^ Furthermore, the depletion of butyrate-producing bacteria favors the expression of Nos2 (an important gene encoding nitrite oxidase synthase), resulting in elevated levels of available nitrate and proliferation of the *Enterobacteriaceae* family, especially *E. coli*.^90^

After fecal microbiota transplantation for CDI treatment, an increase in Bacteroidetes to the detriment of Protobacteria was found.^91^ The important role of Proteobacteria in IBD and CDI diseases is associated with its direct role as a disruptor of intestinal homeostasis and its direct implication in the inflammation of the intestine. The absence of differentiated B-cells and deficiency in the production of specific IgA (specifically targeting Proteobacteria) is correlated with the persistence of Proteobacteria in the inflamed gut.^92^ Other taxonomical alterations related to CDI and IBD are increased levels of *Fusobacterium* and *Mycobacterium* taxa.^93^ The Fusobacteriaceae family has also been found in high proportions in the gut microbiota of patients with CD and UC.^40,75^ *Mycobacterium avium subs. Paratuberculosis* and *Fusobacterium nucleatum* have been recently investigated as potential aggravating factors for IBD.^94^

### *Altered intestinal barrier function and* C. difficile *colonization*

The impairment of intestinal barrier function or disruption of mucosal T cells by inflammatory mediators favor *C. difficile* colonization and toxin production. Some phospholipids, such as phosphatidylcholine and phosphatidylethanolamine, are released during this disruption. Phosphatidylcholine is converted into ethanolamine and glycerol by bacterial phosphodiesterases. *C. difficile* benefits from the breakdown of ethanolamine and utilizes it as a source of nitrogen and carbon.^95,96^ On the other hand, a higher glycosidase activity has been reported in IBD patients than in healthy subjects. Indeed, disruption of intestinal barrier function and the intestinal microbiota also entails the liberation of monosaccharides, which promote the multiplication and colonization of *C. difficile*.^96^ A previous study described in depth how *C. difficile* catabolises microbiota-liberated mucosal carbohydrates and how pathogen expansion is even aided by microbiota-induced elevation of sialic acid levels *in vivo*.^97^

*C. difficile* is able to produce para-cresol (*p*-cresol) through the fermentation of tyrosine in the gut. A recent study demonstrated that this ability provides a competitive advantage over other gut bacteria, including *E. coli, Klebsiella oxytoca*, and *Bacteroides thetaiotaomicron*.^98^ Further studies have proposed that bacterial metabolites, such as *p*-cresol, ammonium, and hydrogen sulfide, notably affect intestinal barrier function and participate in the IBD course.^99^

### Other microbiota signatures and metabolic pathways associated with specific conditions and populations of CDI and IBD

In pediatric population, it has been described that IBD patients with CDI and with a previous history of surgery presented a reduction of *Ruminococcus, Alistipes*, and *Bifidobacterium*.^100^ Even if there are significant differences in the gut microbiota between pediatric patients and adults due to the gut microbiota is not yet fully developed, throughout this review we have observed several discrepancies among the different studies in relation to the presence of some species of *Bifidobacterium* and *Ruminococcus* and their role in the inflamed mucosa (Tables 1 and 2). It has been demonstrated that some strains of *R. gnavus* are able to assimilate mucin monosaccharides, to use sialic acid and to produce propanol and propionate.^101^ As previously described, mucin users are implicated in gut inflammation.^37^ But the finding that not all of *R. gnavus* strains are able to grow on mucin as the sole carbon source^101^ may explain the differences about its depletion or increase in IBD and CDI disease. Further explanations for an overexpression of *Bifidobacterium*,^61,68,102^ *Ruminococcus*,^49,61,67^ or even *Akkermansia*^102^ in the inflamed gut include the important role of the modifications in the intestinal micro-environment, as, for example, an increase of mucus production,^102^ and also microbiota modifications and interactions with aging.

Other three studies have confirmed the reduction of *Alistipes* in pediatric patients with IBD,^37,52,56^ but also in patients with CDI.^102^ *Alistipes* has been associated with protection against CDI and positive modulate the immune response against experimental colitis in mouse models.^103^ Furthermore, it has been proposed as biomarker of CDI,^102^ and used as one of the dominant genera in the fecal bacterial composition of donors for fecal microbiota transplantation to treat CDI, resulting in the successful integration of this bacterial group in the gut ecosystem of the patient.^104^

Finally, and in relation with metabolic pathways, a further analysis showed a reduction in methionine biosynthesis in IBD patients with *C. difficile* after surgery.^100^ Alterations of sulfur and cysteine/methionine metabolism in IBD patients have been previously related to changes in proportions of some bacteria with specific functions involving these pathways,^105^ including *F. prausnitzii* and *Roseburia* among others.^100^ Furthermore, it has been observed that a commercial form of methionine (available as dietary supplement) enhances the viability of *Saccharomyces boulardii* in the gut, especially in acidic environments. This nonpathogenic yeast is classified as a probiotic and it has been used to prevent CDI^106^ and suggested as a treatment of IBD. Furthermore, serological antibodies *Anti-Saccharomyces* have been used as a marker for prediction of CD disease course, within other variables and patient characteristics.^107^

## Microbiota and treatments for IBD and CDI: situation and perspective

As already described in this review, one of the most proposed options in recent years have been fecal microbiota transplantation to restore the altered gut ecosystem. We can find in the literature several studies describing its use in CDI patients with underlying IBD.^108^ In a previous study treating patients with CDI and concurrent IBD, the effectiveness of FMT was between 79% and 88%, after one and two interventions, respectively.^109^ A further study also reported the efficacy of FMT to treat recurrent CDI in IBD, but authors found that more than half of patients required IBD treatment escalation shortly after FMT.^110^ Similarly, in the study of Khoruts et al.^111^ results showed that FMT was less effective in IBD patients suffering recurrent CDI than in those without IBD, as more than 25% of the studied IBD patients have a disease flare following FMT, especially in those cases with extensive colon involvement, and they required a treatment with prednisone. Hypothesis about the problems with FMT in these patients include implantation of the major taxa in the gut and deficiency in host immune defenses.^111^ Meighani et al.^112^ found a good response to FMT in patients with CDI and IBD. In their study, three patients who failed therapy had newly diagnosis of IBD and one presented severe active disease. Therefore, authors conclude that FMT is a good alternative treatment for well-controlled IBD patients with recurrent CDI.

Consistent with the microbiota changes observed in IBD and CDI patients, a specific microbiota signature for fecal microbiota donors has been described^80,113^ (Table 3). The selection of microbes has important underlying metabolic mechanisms, such as the production of butyrate in the gut, as largely described in the section above.

**Table 3.** Group of bacteria modifications associated with CDI and IBD. Main characteristics and role in disease and fecal microbiota transplant treatment.

Bacteria group at genus level	Taxonomy	Main characteristics and role in the intestinal metabolic activity◊	Gut ecosystem modifications observed	Fecal microbiota transplant treatment	
				Expected changes after taxa restoration	Main microbiota communities identified in bacteriotherapy studies for gut restoration (∇)
*Alistipes*	Bacteroidetes Bacteroidia Bacteroidales Rikenellaceae	Gram-stain negative Straight or slightly curved rods Non-spore forming Non-motile Obligately anaerobic Produce succinic acid (major glucose metabolic end product) and acetic acid (minor) Produce indole and digest gelatin Bile tolerant	Depletion in children with both CDI and IBD^100^	– Stable engraftment and restoration of the structure of the gut microbiota. Cessation of CD-related changes and resolution of other gastrointestinal symptoms (in combination with genera *Bacteroides* and *Parabacteroides*)^104^	*Alistipes* and *Blautia* (positively correlated with colonic melatonin receptor expression) (AM)^114^
*Blautia*	Firmicutes Clostridia Clostridiales Lachnospiraceae	Gram-stain-positive, non-motile coccoid or oval-shaped short rods Obligate anaerobe Growth is stimulated by fermentable carbohydrates. End products after fermentation include acetate, ethanol, lactate, butyrate, and succinate - Some species also produce bile salt hydrolase (*B. obeum*)	Depletion in adult patients with both CDI and IBD^76^	- Increase of butyrate production (negative correlation with the presence of *C. difficile*)^81^ - Restitution of microbiota bile salt hydrolases (restoration of gut bile metabolism)^87^	*Blautia Bacteroides and Ruminococcus* (in detriment of *Enterococcus, Escherichia, Shigella*) (CDI)^81^ *Blautia producta, Escherichia coli, Clostridium bifermentans* (to antagonize *C. difficile* and restore *Bacteroides levels*) (CDI)^82^ *Blautia* and Ruminococcaceae (associated to colonization resistance) (CDI)^83^ *Blautia hansenii* (protective against infection) (CDI)^83^ *Blautia, Coprococcus, Faecalibacterium* (restoration after FMT) (CDI)^84^ *Blautia* and *Blautia producta* (Taxa used in bacteriotherapy studies) (CDI)^85^ *Blautia* and *Alistipes* (positively correlated with colonic melatonin receptor expression)^114^ Blautia, *Dorea, Roseburia, Oscillobacter* (CDI)^115^
*Dorea*	Firmicutes Clostridia Clostridiales Lachnospiraceae	Gram-stain-positive rods, non-spore forming, non-motile. Obligately anaerobic and chemo-organotrophic. Major end products of glucose metabolism include ethanol, formate, acetate, H_2_, and CO_2_	Depletion in adult patients with both CDI and IBD^76^	- Recovery of short fatty acid production and therefore the metabolic activity of the microbial community (related with CDI remission)^115^	*Dorea* (CDI)^85^ *Dorea, Blautia Roseburia, Oscillobacter* (CDI)^115^
*Clostridium*	Firmicutes Clostridia Clostridiales Clostridiaceae	Usually Gram-stain-positive rods. Motile or non-motile (when motile, cells usually are peritrichous) The majority of species form oval or spherical endospores that usually distend the end.	Increase in the gut of patients with CDI and IBD^76^ (although altered patterns of *Clostridium* group are not always the same in CDI and IBD patients separately)	- Restore the phylogenetic richness of the gut (restoration of Firmicutes/Bacteroidetes ratio)^115^ - Increase of butyrate production (negative correlation with the presence of *C. difficile*)^81^	*Clostridium bifermentans, Clostridium innocuum, Clostridium ramosum*, *Clostridium cocleatum*^85^ *Blautia producta, Escherichia coli, Clostridium bifermentans* (to antagonize *C. difficile* and restore *Bacteroides levels*) (CDI)^82^
*Enterococcus*	Firmicutes Bacilli Lactobacillales Enterococcaceae	Gram-stain-positive rods, non-spore forming. Some strains motile by scanty flagella Cells are ovoid Facultative anaerobic Carboxyphilic (C0_2_ dependant)	Increase in the gut of patients with both CDI and IBD^76^ (although altered patterns of *Enterococcus* group are not always the same in CDI and IBD patients studied separately)	- Reduction of lactic acid-producing bacteria and their metabolites - Restitution of the intestinal homeostasis	*Enterococcus faecalis* (CDI)^85^
*Faecalibacterium*	Firmicutes Clostridia Clostridiales Ruminococcaceae	Usually Gram-stain-negative Rod-shaped cells Non-motile Non-sporulating Butyrate production Metabolize pectin, uronic acids, and N-acetyl glucosamine	Depletion in children with both CDI and IBD^100^	- Increase methionine biosynthesis to improve intestinal antioxidant capacity^105^ - Increase the anti-inflammatory response in the gut^49^	*Faecalibacterium prausnitzii* and *Bacteroides ovatus* (CDI)^115^ *Faecalibacterium, Blautia, Coprococcus* (CDI)^84^
*Roseburia*	Firmicutes Clostridia Clostridiales Lachnospiraceae	Gram-stain-negative to variable stain reaction Rod-shaped cells Non-sporulating Motile (37ºC) by flagella Chemo-organotrophic Strictly anaerobic Use of carbohydrates as a carbon and energy source Produces H_2,_ CO_2_ and large amounts of butyrate after fermentation of glucose and acetate May produce lactate, formate, and ethanol	Depletion in children with both CDI and IBD^100^	- Increase methionine biosynthesis to improve intestinal antioxidant capacity^105^ - Increase of butyrate production (negative correlation with dysbiosis in UC)^74^	*Roseburia, Oscillobacter, Blautia, Dorea* (CDI)^115^
*Ruminococcus gnavus*	Firmicutes Clostridia Clostridiales Ruminococcaceae	Gram-stain positive cell wall structure (but many stain Gram-negative) Cells are coccoid, in pairs and chains Only few motile (flagella) Chemo-organotrophic Strictly anaerobic Growth is stimulated by fermentable carbohydrates and the end products include acetate, formate, ethanol, lactate, and succinate Mucin degradation	Increase of *Ruminococcus gnavus* in adult patients with both CDI and IBD^76^ Depletion (genus level) in children with both CDI and IBD^100^	- Reduction of digestive endogenous mucin substrate to prevent other bacteria proliferation and to allow host bacteria to multiply^67^	*Ruminococcus, Blautia, Bacteroides* (in detriment of *Enterococcus, Escherichia, Shigella*) (CDI)^81^ Ruminococcaceae, *Blautia* (associated to colonization resistance) (CDI)^83^

**◊** According to Bergey´s Manual of Systematics of Archea and Bacteria.

**∇** Specific studies addressing the efficacy of different bacterial species in the restoration of the gut microbiota after inflammation and/or infection.

**CDI**: *Clostridium difficile* infection.

**AM**: animal model.

A previous study selected a total of 37 bacteria to treat dysbiosis during CDI, which could be administrated orally in a noninvasive way.^85^ In this contest, some problems derived from FMT are related to the actual method of the feces delivery, which may require colonoscopy and sedation.^116^ In addition, other problems could include the degree of engraftment and immune response to the transplanted microbiota (donor-recipient incompatibilities), stemming from an underlying genetic factor.^80^ A previous study suggested only a marginal risk of worsening in FMT-treated IBD patients, and hypothesized the role of donors to induce remission or to induce worsening in IBD activity.^117^ Other described complications include the transmission of parasites from donors to patients by FMT, but without gastrointestinal symptomatology.^118^ A recent study used washed microbiota transplantation in mice and concluded that the technique avoids the virus transmission among other complications and is safer than crude FMT.^119^

In addition to FMT, probiotic nutrition with multiple strains for gastrointestinal health modulation has been proposed as an effective and safe treatment.^120^ One recent study proposed the strain *Bacillus licheniformis* to treat colitis, which seems to modulate the gut microbiota composition and has been associated with a decrease in Bacteroidetes.^121^ Other probiotics classically used in different trials to reduce intestinal inflammation are *Lactobacillus rhamnosus, Lactobacillus plantarum, Lactobacillus acidophilus*, and *Enterococcus faecium*. However, the available results on their effectiveness in both CD and UC are still not concise.^122^ To prevent the likelihood of incurring CDI, a combination of various probiotics, including *Streptococcus faecalis, Bacillus mesentericus*, and *Clostridium butyricum*,^123^ has been proposed (once again, we can find among “protective” strains those associated with butyric acid production). Competition for the niche with non-toxigenic *C. difficile* strains has also been suggested for CDI prevention. *Bacillus clausii* and *Lactobacillus reuteri* also act as probiotics for this infection because they secrete compounds that directly inhibit *C. difficile*.^124^ Those probiotics proposed for CDI and IBD separately could be used jointly to treat both diseases. However, a recent study underlines the important role of mutual interaction of probiotics, which can inhibit other probiotics or protective taxa in the gut.^125^ Therefore, further studies addressing these metabolic interactions are necessary to better understand the role of these probiotics in both diseases.

## Conclusions and future directions

In this review, we have summarized the gut microbiota changes associated with both forms of IBD and CDI and their similarity with the dysbiosis that occurs in the CDI. IBD is itself a complicated and poorly understood disease. The alteration of the microbiota and the metabolic environment of the gut have direct consequences in chronic inflammation and in the colonization and multiplication of opportunistic pathogens, with *C. difficile* being one of the most important causes of infection in this group. Our analysis reveals important modifications in specific taxa that recur in both diseases despite the intrinsic differences of each study (variable environment, genetic diversity, medication usage, smoking history, and variable diet). Furthermore, the investigation of the metabolic pathways of these groups of bacteria reveals the specific mechanism of action in the epithelial cells and lumen in the gut. Elucidating the impact of bacterial metabolites in other microbial communities, it is possible to better discern between protective bacteria and those that cause harm. All the advances in new sequencing technologies have provided a large number of publications that apply these methods to better understand intestinal inflammation. However, in this review, we highlight that there is an insufficient number of studies addressing the microbiota composition and its changes in the gut of patients suffering from both CDI and IBD. Furthermore, the only available data are focused either on the epidemiology and treatment of the infection in IBD patients or on the microbiota composition of adult patients, but there are no results on other patient groups, such as the elderly, pediatric, or pregnant IBD populations. In addition, the results regarding adults are scarce and supported by only a few studies. Therefore, there is an urgent need to develop new research lines addressing the changes in the gut microbiota in IBD patients suffering from CDI. These studies will provide results that are now necessary to develop new therapeutic strategies to prevent and treat *C. difficile* and its infection in IBD.
